# Self-reported hypertension in adults: a cross-sectional study using VIGITEL data, southern region of Brazil, 2023

**DOI:** 10.1590/S2237-96222026v35e20250626.en

**Published:** 2026-08-28

**Authors:** Eduardo Augusto Bruzamarello, Patrícia Alessandra Carneiro, Claudicéia Risso Pascotto, Franciele Ani Caovilla Follador, Lirane Elize Defante Ferreto

**Affiliations:** 1 Universidade Estadual do Oeste do Paraná, Programa de Pós-graduação em Ciências Aplicadas à Saúde, Francisco Beltrão, PR, Brazil Universidade Estadual do Oeste do Paraná Universidade Estadual do Oeste do Paraná Programa de Pós-graduação em Ciências Aplicadas à Saúde Francisco Beltrão PR Brazil

**Keywords:** Chronic Disease, Cardiovascular Diseases, Risk Factorebrs, Health Surveys, Cross-Sectional Studies, Enfermedad Crónica, Enfermedades Cardiovasculares, Factores de Riesgo, Encuestas Epidemiológicas, Estudios Transversales.

## Abstract

**Objectives:**

To estimate prevalence of self-reported hypertension and to analyze associated factors in adults living in the state capital cities of the Southern region of Brazil in 2023.

**Methods:**

This was a cross-sectional study with adults (≥18 years) from the cities of Curitiba, Florianópolis and Porto Alegre, using data from the 2023 Chronic Disease Risk and Protective Factors Surveillance Telephone Survey (VIGITEL). The variables were hypertension, sociodemographic factors, health indicators and lifestyle. Statistical analysis used a complex sampling design with weighting. Bivariate analyses and multivariate Poisson regression were performed to estimate prevalence ratios (PR) and confidence intervals (95%CI).

**Results:**

The sample totaled 2,255 individuals. Hypertension prevalence was 28.0% (95%CI 25.4; 30.8) and ranged from 22.7% (95%CI 19.4; 26.5) in Florianópolis to 32.8% (95%CI 28.2; 37.8) in Porto Alegre. In the final multivariate analysis, the factors that remained independently associated with hypertension were increasing age (PR 13.15; 95%CI 5.93; 29.15 for ≥70 years), obesity (PR 2.01; 95%CI 1.70; 2.38), and diabetes (PR 1.53; 95%CI 1.31; 1.79).

**Conclusion:**

Hypertension prevalence in the state capital cities of the Southern region of Brazil was 28% and differed between the cities. The main associated factors were increasing age, obesity and diabetes. The findings emphasized the importance of prevention strategies focused on modifiable risk factors and monitoring of the most vulnerable groups.

## Resumen

**Objetivos:**

Estimar la prevalencia de hipertensión autoinformada y analizar los factores asociados en adultos residentes en las capitales de la región sur de Brasil en 2023.

**Métodos:**

Estudio transversal con adultos (≥18 años) de Curitiba, Florianópolis y Porto Alegre, utilizando datos del Sistema de Vigilancia de Factores de Riesgo y Protección para Enfermedades Crónicas por Encuesta Telefónica (Vigitel) 2023. Las variables fueron hipertensión, factores sociodemográficos, indicadores de salud y estilo de vida. El análisis estadís-tico empleó un diseño de muestreo complejo con ponderación. Se realizaron análisis bivariados y regresión de Poisson multivariada para estimar las razones de prevalencia (RP) y los intervalos de confianza (IC95%).

**Resultados:**

La muestra totalizó 2.255 individuos. La prevalencia de hipertensión fue del 28,0% (IC95%: 25,4; 30,8) y osciló entre el 22,7% (IC95%: 19,4; 26,5) en Florianópolis y el 32,8% (IC95%: 28,2; 37,8) en Porto Alegre. En el análisis multivariante final, los factores que permanecieron asociados de forma independiente con la hipertensión fueron el aumento de la edad (PR 13,15; IC95%: 5,93; 29,15 para ≥70 años), la obesidad (PR 2,01; IC95%: 1,70; 2,38) y la diabetes (PR 1,53; IC95%: 1,31; 1,79).

**Conclusión:**

La prevalencia de hipertensión en las capitales de la región sur del país fue del 28% y varió entre ciudades. Los principales factores asociados fueron la edad, la obesidad y la diabetes. Los hallazgos refuerzan la importancia de las estrategias de prevención centradas en los factores de riesgo modificables y el seguimiento de los grupos más vulnerables.

Ethical aspects This research used anonymized, public-domain databases.

## Introduction

In 2019, hypertension affected 1.3 billion people worldwide [1]. In the same year, in Brazil, 38.1 million (23.9%) adults over 18 years of age reported having hypertension; of these, 26.4% were women and 21.1% were men. That data showed that the percentage of affected people increases proportionally with age, reaching 62.1% in people aged 75 or older [2]. Hypertension is diagnosed when blood pressure levels are ≥140 mmHg systolic and/or ≥90 mmHg diastolic, a condition that, if not treated properly, becomes a risk factor for cardiovascular diseases [3].

Cardiovascular diseases, as well as increased blood pressure, are associated with risk factors related to lifestyle, genetic predisposition and advancing age [4]. Due to the proportion of the population with hypertension, in 2006, the Chronic Disease Risk and Protective Factors Surveillance Telephone Survey (*Sistema de Vigilância de Fatores de Risco e Proteção para Doenças Crônicas por Inquérito Telefônico,* VIGITEL) was implemented to monitor, collect and disseminate health indicators, being conducted in the 26 Brazilian state capital cities and Federal District [5]. This approach makes it possible to compare individuals with and without altered blood pressure, which enables understanding of the lifestyle habits and health profile of the population and favors identification of potential targets for public health intervention.

Given that the Southern region had the highest proportion of elderly people in Brazil in 2022 [6] and that aging is a determining factor regarding increasing blood pressure levels [4], it is noteworthy that the Southern states of Brazil concentrated 24.5% of people with hypertension in Brazil in 2019 [2]. This makes it relevant to investigate the main risk factors associated with the development of this disease, in order to improve strategies for controlling related complications.

The objective of this study was to estimate self-reported prevalence of hypertension and to analyze associated factors among adults living in the three state capital cities of the Southern region of Brazil in 2023.

## Methods

## Study design

This is a cross-sectional study based on publicly available secondary data.

### Setting

This study analyzed data on residents of the capital cities of the three states that comprise the Southern region of Brazil, namely Paraná, Santa Catarina and Rio Grande do Sul. The data was derived from the VIGITEL system, referring to 2023 [7], and was accessed on April 28, 2025.

### Participants

The inclusion criteria were: individuals aged 18 years or older, residing in Curitiba, Florianópolis and Porto Alegre. Individuals with missing data (i.e., answers such as “don’t know” or “prefer not to answer”) for any of the variables of interest were excluded.

### Variables

The outcome variable was hypertension, determined by an affirmative answer to the question: “Has a doctor ever told you that you have high blood pressure?”. The independent variables were divided as follows:

1. Sociodemographic: sex (male, female), age group (18-29, 30-39, 40-49, 50-59, 60-69 and ≥70 years), current marital status (single, legally married, domestic partnership for more than six months, widowed, separated or divorced), schooling (no schooling, incomplete elementary, complete elementary, complete high school, bachelor’s degree or higher) and race/color (White, Black, mixed-race, Asian, Indigenous);
2. Health indicators: self-reported diagnosis of diabetes (yes, no) and obesity (body mass index - BMI≥30.0 kg/m² [8]);
3. Lifestyle: tobacco smoker (yes, no) and sedentary lifestyle (defined as physical inactivity in the last three months). The variable related to alcohol consumption was excluded from the analyses due to 12.5% of inconsistent or missing answers.

### Data sources and measurement

The survey used a dual baseline design, combining random sampling of landline and mobile telephone lines. The detailed sampling, respondent selection and data weighting methodology is described in the literature [5]. The data collection method consisted of telephone interviews using computers, whereby the questions were read on a monitor, and the answers were recorded directly electronically [5].

### Bias

Data weighting was used in the statistical analysis in order to mitigate potential selection and non-response biases. The researchers acknowledge the potential for information (or measurement) bias, as the variables were self-reported.

### Study size

The final sample consisted of 2,255 individuals who met the inclusion and exclusion criteria described.

### Statistical methods

Hypertension prevalence and its covariates were estimated using proportions, considering the survey’s complex sampling design.

Initially, descriptive analyses were performed to characterize the sample, followed by bivariate analyses using the Rao-Scott test of independence (F), this being an adjusted version of Pearson’s chi-square test, appropriate for complex sampling data [9].

In order to investigate factors associated with hypertension in a multivariate manner, controlling for possible confounding factors, Poisson regression with robust variance for complex samples was employed [10]. Adjusted prevalence ratios (PR) and their respective 95% confidence intervals (95%CI) were estimated. The final multivariate model included all investigated variables of interest.

Descriptive and bivariate analyses were performed using IBM SPSS Statistics software, version 27.0, using the complex samples module. Multivariate analysis (Poisson regression) was conducted using the R software (version 4.5.1) survey package.

Population expansion weighting (pesorake) was applied in both analyses. This weighting, provided in the database, was calculated by VIGITEL itself using a post-stratification method (rake). The pesorake simultaneously corrected for the different probability of selection of each individual and sampling distortions resulting from non-response and coverage, adjusting the sample so that it corresponded to the population estimates taken from the Demographic Census for each state capital city, according to sex, age group and level of schooling strata.

Association with a p-value<0.050 was considered statistically significant.

## Results

The study sample consisted of 2,255 adults residing in the capital cities of the Southern region of Brazil. Weighted prevalence of self-reported hypertension for the three capital cities combined was 28.0% (95%CI 25.4; 30.8). There was statistically significant variation between the municipalities. Porto Alegre presented a 32.8% prevalence rate (95%CI 28.2; 37.8), while Florianópolis had the lowest prevalence, at 22.7% (95%CI 19.4; 26.5) (Table 1).

**Table 1:** Self-reported hypertension prevalence and 95% confidence intervals (95%CI), by Southern region state capital cities. Curitiba, Florianópolis and Porto Alegre, 2023 (n=2,255)

Municipality^a^	N^b^	Hypertension prevalence (%) (95%CI)^c^
Curitiba	757	25.6 (22.0; 29.6)
Florianópolis	756	22.7 (19.4; 26.5)
Porto Alegre	742	32.8 (28.2; 37.8)
Total	2,255	28.0 (25.4; 30.8)

^a^Rao-Scott test of independence (F) for statistical difference between the municipalities (F 5.68; p-value 0.007); ^b^The (N) values refer to unweighted sample counts; ^c^The prevalence rates are weighted.

A predominance of females was observed (n=1,385; 61.4%) (Table 2). The most predominant age group was 70 years or older (n=468; 20.8%). Most respondents reported being legally married (n=870; 38.6%). There was a predominance of White race/color (n=1,724; 76.5%). Regarding education level, the highest frequency was found for individuals with a bachelor’s degree or higher (n=941; 41.7%).

In the bivariate analysis (Table 2), hypertension prevalence increased progressively as age increased (F 34.29; p-value<0.001). Current marital status (F 14.8; p-value<0.001) also showed statistically significant association, with hypertension being more prevalent among widows/widowers, at 58.8% (95%CI 47.4; 69.5). Hypertension prevalence decreased progressively as education level increased (F 21.24; p-value<0.001).

**Table 2:** Hypertension prevalence, confidence interval (95%CI) and bivariate analysis, by sociodemographic characteristics. Curitiba, Florianópolis and Porto Alegre, 2023 (n=2,255)

Variable	Category	N^a^	Hypertension prevalence (%) (95%CI)^b^	p-value^c^
**Sex**	Male	870	28.1 (24.3; 32.3)	0.943
	Female	1.385	27.9 (24.5; 31.6)	
**Age group (years)**	18-29	262	4.4 (2.0; 9.2)	<0.001
	30-39	315	19.1 (12.9; 27.5)	
	40-49	381	23.3 (17.7; 30.1)	
	50-59	399	38.8 (32.8; 45.1)	
	60-69	430	48.2 (41.7; 54.9)	
	70+	468	66.3 (60.4; 71.7)	
**Current marital status**	Single	625	17.2 (13.1; 22.1)	<0.001
	Legally married	870	32.4 (28.5; 36.6)	
	Domestic partnership for more than six months	317	29.3 (22.4; 37.2)	
	Widowed	244	58.8 (47.3; 69.5)	
	Separated or divorced	199	39.4 (29.8; 49.9)	
**Race/color**	White	1.724	28.7 (25.6; 31.9)	0.825
	Black	129	28.2 (17.5; 42.1)	
	Asian	22	32.9 (14.5; 58.6)	
	Mixed-race	368	25.1 (19.8; 31.2)	
	Indigenous	12	35.1 (11.5; 69.1)	
**Schooling**	No schooling/incomplete elementary	201	53.1 (42.7; 63.2)	<0.001
	Complete elementary	363	45.2 (37.8; 52.8)	
	Complete high school	750	26.0 (21.7; 30.8)	
	Bachelor’s degree or higher	941	20.0 (16.7; 23.9)	

^a^The (N) values refer to unweighted sample counts; ^b^The prevalence rates are weighted; ^c^Rao-Scott test of independence (F).

The results of the multivariate analysis, adjusted for covariates, are shown in Table 3. After adjustment, the following remained as factors independently associated with hypertension: increasing age (PR 13.15; 95%CI 5.93; 29.15 for ≥70 years), obesity (PR 2.01; 95%CI

1.70; 2.38) and diabetes (PR 1.53; 95%CI 1.31; 1.79).

**Table 3:** Adjusted prevalence ratios (PR) and confidence intervals (95%CI) for hypertension, according to sociodemographic, health indicator and lifestyle variables. Curitiba, Florianópolis and Porto Alegre, 2023 (n=2,255)

Variable	Category	PR (95%CI)^a^	p-value
**Sociodemographic**			
Sex	Male	1	
	Female	1.08 (0.91; 1.27)	0.377
Age group (years)	18-29	1	
	30-39	4.27 (1.83; 9.97)	<0.001
	40-49	4.74 (2.10; 10.68)	<0.001
	50-59	7.81 (3.54; 17.25)	<0.001
	60-69	9.24 (4.14; 20.61)	<0.001
	70+	13.15 (5.93; 29.15)	<0.001
Current marital status	Single	1	
	Legally married	0.91 (0.70; 1.17)	0.450
	Domestic partnership for more than six months	1.09 (0.78; 1.52)	0.609
	Widowed	1.02 (0.76; 1.38)	0.901
	Separated or divorced	1.11 (0.81; 1.51)	0.531
**Race/color**	White	1	
	Black	1.21 (0.79; 1.87)	0.378
	Asian	1.46 (0.80; 2.65)	0.213
	Mixed-race	0.91 (0.73; 1.12)	0.358
	Indigenous	0.90 (0.49; 1.66)	0.743
Schooling	No schooling/incomplete elementary	1	
	Complete elementary	1.15 (0.91; 1.43)	0.237
	Complete high school	0.99 (0.79; 1.25)	0.944
	Bachelor’s degree or higher	0.79 (0.61; 1.01)	0.064
**Health indicators**			
Obese	Not obese	1	
	Obese	2.01 (1.70; 2.38)	<0.001
Diabetes	Not diabetic	1	
	Diabetic	1.53 (1.31; 1.79)	<0.001
**Lifestyle**			
Sedentary	Not sedentary	1	
	Sedentary	1.13 (0.95; 1.35)	0.153
Tobacco smoker	Non-smoker	1	
	Smoker	0.97 (0.75; 1.25)	0.808

^a^Adjusted for all variables listed in the table.

## Discussion

In this study, it was possible to identify that hypertension among adults residing in the state capital cities of the Southern region of Brazil is associated with being older, lower schooling, obesity, sedentary lifestyle and diabetes. However, self-reported hypertension prevalence among adults in these capital cities was similar to that found in other studies, also using VIGITEL data, but for 2019 [11].

This study had limitations, such as use of secondary self-reported data, which are subject to recall bias, incomplete data, underreporting and exclusion of individuals without access to a telephone, which may limit the sample size.

Answer prevalence was higher among females, although no statistically significant differences were found between the sexes. This data is consistent with the global epidemiological scenario, in which approximately 600 million women suffer from altered blood pressure levels, a condition indicated as the main cause of female mortality [12].

The highest self-reported hypertension prevalence was concentrated in those aged 70 years and older. Age has been identified as a non-modifiable risk factor, characterized by an increasing probability of developing high blood pressure over time, especially after the fifth decade of life. This increase is linked to physiological and hemodynamic changes resulting from aging [13], with a projected 7% growth in global prevalence between 2015 and 2050 [14]. Therefore, older individuals have greater difficulty adopting new habits and establishing a healthier lifestyle, with limitations in understanding the impact and severity of the disease, which hinders incorporation of self-care practices [15].

Regarding modifiable criteria, predominance of hypertension was identified in people with no schooling or with incomplete elementary education, pointing to schooling as an influence on health inequality [16]. It has been observed that individuals with lower schooling are more vulnerable to developing hypertension, an event that is potentially associated with poorer socioeconomic conditions, which may lead to difficulty in accessing health services [17].

Regarding daily lifestyle habits, considered modifiable factors, such as physical exercise, healthy diet and not consuming tobacco and alcohol, these are the main non-pharmacological control actions, as they help improve cardiovascular health [18]. Non-adherence to physical activity leads to sedentary behavior, which causes an increase in body fat and accumulation in vein and artery lumen, generating arteriosclerosis that raises blood pressure levels [19]. Another consequence is obesity, prevalent among the hypertensive individuals studied, which triggers an increase in blood pressure due to alterations in cardiometabolic mechanisms, increases cardiac output and, consequently, dysregulates systemic cardiovascular resistance [20].

It was also found that hypertension prevalence was lower among smokers, although this was not statistically significant in this study. Smoking has been identified as the second leading risk factor directly affecting cardiovascular diseases, in addition to contributing to the development of dyslipidemia and diabetes [21]. Given that diabetes causes glucose dysregulation in the body, when combined with hypertension, and that this characteristic was evident in the study [22], high risk of complications such as chronic kidney disease, retinopathy and sexual dysfunction stood out [23].

In summary, hypertension prevalence in the population of the South of Brazil was 28%, linked to lifestyle habits such as sedentary behavior (correlated with obesity, diet and alcohol and tobacco consumption), schooling, as well as non-modifiable factors such as age. These findings highlighted the importance of public policies aimed at promoting healthy habits, providing tools for controlling modifiable risk factors and preventing worsening of non-modifiable risk factors.

## Data Availability

This study used public domain data, available at the Ministry of Health’s official repository: https://svs.aids. gov.br/download/Vigitel/. Codes used to build variables that are not available in the original database can be obtained by request from the corresponding author.
