# Supplementary material for: Self-reported hypertension in adults: a cross-sectional study using VIGITEL data, southern region of Brazil, 2023
Source: Epidemiol Serv Saude. 2026 Aug 28;35:e20250626. doi: 10.1590/S2237-96222026v35e20250626.en (PMC13686388; doi:10.1590/S2237-96222026v35e20250626.en)
Supplement: Translation

# Translation: Hipertensão autorreferida em adultos: estudo transversal com dados do Vigitel, região Sul, 2023

**DOI:** 10.1590/S2237-96222026v35e20250626.pt

**Authors:** Eduardo Augusto Bruzamarello, Patrícia Alessandra Carneiro, Claudicéia Risso Pascotto, Franciele Ani Caovilla Follador, Lirane Elize Defante Ferreto

**Affiliations:** 1 Universidade Estadual do Oeste do Paraná, Programa de Pós-graduação em Ciências Aplicadas à Saúde, Francisco Beltrão, PR, Brazil Universidade Estadual do Oeste do Paraná Universidade Estadual do Oeste do Paraná Programa de Pós-graduação em Ciências Aplicadas à Saúde Francisco Beltrão PR Brazil

**Keywords:** Doença Crônica, Doenças Cardiovasculares, Fatores de Risco, Inquéritos Epidemiológicos, Estudos Transversais

## Abstract

**Objetivos:**

Estimar a prevalência de hipertensão autorreferida e analisar os fatores associados em adultos residentes nas capitais da região Sul do Brasil em 2023.

**Métodos:**

Estudo transversal com adultos (≥18 anos) de Curitiba, Florianópolis e Porto Alegre, utilizando dados do Sistema de Vigilância de Fatores de Risco e Proteção para Doenças Crônicas por Inquérito Telefônico (Vigitel) 2023. As variáveis foram hipertensão, fatores sociodemográficos, indicadores de saúde e estilo de vida. A análise estatística utilizou o plano amostral complexo, com ponderação. Foram realizadas análises bivariadas e regressão de Poisson multivariada para estimar as razões de prevalência (RP) e os intervalos de confiança (IC95%).

**Resultados:**

A amostra totalizou 2.255 indivíduos. A prevalência de hipertensão foi de 28,0% (IC95% 25,4; 30,8) e variou de 22,7% (IC95% 19,4; 26,5) em Florianópolis a 32,8% (IC95% 28,2; 37,8) em Porto Alegre. Na análise multivariada final, os fatores que permaneceram independentemente associados à hipertensão foram aumento da faixa etária (RP 13,15; IC95% 5,93; 29,15 para ≥70 anos), obesidade (RP 2,01; IC95% 1,70; 2,38) e diabetes (RP 1,53; IC95% 1,31; 1,79).

**Conclusão:**

A prevalência de hipertensão nas capitais da região Sul do país foi de 28% e diferiu entre as cidades. Os principais fatores associados foram faixa etária, obesidade e diabetes. Os achados reforçaram a importância de estratégias de prevenção focadas nos fatores de risco modificáveis e no monitoramento dos grupos de maior vulnerabilidade.

Aspectos éticos Esta pesquisa utilizou bancos de dados de domínio público e anonimizados.

## Introdução

Em 2019, a hipertensão afetava 1,3 bilhão de pessoas no mundo [1]. No mesmo ano, no Brasil, 38,1 milhões (23,9%) de adultos com mais de 18 anos relataram ter hipertensão; destes, 26,4% foram mulheres, e 21,1% homens. Isso evidenciou que a porcentagem aumenta proporcionalmente com o avançar da idade, chegando a 62,1% em pessoas com 75 anos ou mais [2]. O diagnóstico é estabelecido quando os níveis pres-sóricos apresentam valores ≥140 mmHg de pressão arterial sistólica e/ou valores ≥90 mmHg de pressão arterial diastólica, condição que, se não tratada de forma adequada, torna-se fator de risco para doenças cardiovasculares [3].

Esse quadro de patologias cardíacas, assim como o aumento da pressão, está associado a fatores de risco relacionados ao estilo de vida, à predisposição genética e ao avanço da idade [4]. Devido à proporção da população com hipertensão, em 2006, o Sistema de Vigilância de Fatores de Risco e Proteção para Doenças Crônicas por Inquérito Telefônico (Vigitel) foi implementado para monitorar, coletar e divul-gar indicadores de saúde, realizado nas 26 capitais brasileiras e no Distrito Federal [5]. Tal abordagem possibilita comparar indivíduos com e sem alteração de pressão, o que permite compreender os hábitos de vida e o perfil de saúde da população e favorece a identificação de potenciais alvos de intervenção em saúde pública.

Considerando que a região Sul possuía a maior proporção de idosos do país em 2022 [6] e que o envelhecimento é fator determinante para aumentar os níveis pressóricos [4], destaca-se que os estados do Sul concentravam 24,5% das pessoas com hiper-tensão do Brasil em 2019 [2]. Tornou-se relevante investigar os principais fatores de risco associados ao desenvolvimento dessa doença, a fim de aprimorar as estratégias de controle dos agravos relacionados.

Este estudo teve como objetivos estimar a prevalên-cia de hipertensão autorreferida e analisar os fatores associados em adultos residentes nas capitais da região Sul do Brasil em 2023.

## Métodos

### Delineamento do estudo

Trata-se de estudo transversal, com base em dados secundários de acesso público.

### Contexto

O estudo analisou dados de residentes das capitais do Paraná, de Santa Catarina e do Rio Grande do Sul, da região Sul do Brasil. Os dados são provenientes do sistema Vigitel, referentes a 2023 [7], e foram acessados em 28 de abril de 2025.

### Participantes

Os critérios de inclusão foram: indivíduos com idade igual ou superior a 18 anos, residentes em Curitiba, Florianópolis e Porto Alegre. Foram excluídos os indi-víduos com dados ausentes (ou seja, respostas do tipo “não sabe” ou “não quis responder”) para qualquer uma das variáveis de interesse.

### Variáveis

A variável desfecho foi a hipertensão, determinada pela resposta afirmativa à pergunta: “Algum médico já lhe disse que tem pressão alta?”. As variáveis independentes foram divididas conforme a seguir.

1. Sociodemográficas: sexo (masculino, feminino), faixa etária (18-29, 30-39, 40-49, 50-59, 60-69 e ≥70 anos), estado conjugal atual (solteiro, casado legalmente, união estável há mais de seis meses, viúvo, separado ou divorciado), escolari-dade (sem instrução, fundamental incompleto, fundamental completo, ensino médio completo, superior completo ou mais) e raça/cor (branca, preta, parda, amarela, indígena).
2. Indicadores de saúde: diagnóstico de diabetes autorreferido (sim, não) e obesidade (índice de massa corporal - IMC≥30,0 kg/m² [8]).
3. Estilo de vida: tabagismo (sim, não) e sedentaris-mo (definido como inatividade física nos últimos três meses). A variável relacionada ao consumo de álcool foi excluída das análises em razão de apresentar 12,5% de respostas inconsistentes ou não informadas.

### Fonte de dados e mensuração

O inquérito utilizou um delineamento de base dupla, combinando sorteio de linhas de telefonia fixa e móvel. A metodologia detalhada da amostragem, a seleção de entrevistados e a ponderação dos dados encon-tram-se descritas na literatura [5]. O método de coleta consistiu em entrevistas telefônicas com o emprego de computadores, nas quais as perguntas eram lidas em monitor, e as respostas registradas diretamente em meio eletrônico [5].

### Viés

Para diminuir potenciais vieses de seleção e de não resposta, foi utilizada a ponderação dos dados na análise estatística. Reconheceu-se, ainda, o potencial de viés de informação (ou aferição), pois as variáveis foram autorreferidas.

### Tamanho do estudo

A amostra final foi composta por 2.255 indivíduos que atenderam aos critérios de inclusão e exclusão descritos.

### Métodos estatísticos

As prevalências de hipertensão e de suas covariáveis foram estimadas por meio de proporções, consideran-do-se o plano amostral complexo do inquérito.

Inicialmente, foram realizadas análises descritivas para caracterizar a amostra, seguidas de análises bivariadas por meio do teste de independência de Rao-Scott (F), uma versão ajustada do teste do qui-quadrado de Pearson, apropriada para dados de amostragem complexa [9].

Para investigar os fatores associados à hipertensão de forma multivariada, controlando para possíveis fato-res de confusão, foi empregada a regressão de Poisson com variância robusta para amostras complexas [10]. Foram estimadas as razões de prevalência (RP) ajusta-das e seus respectivos intervalos de confiança de 95% (IC95%). O modelo multivariado final incluiu todas as variáveis de interesse investigadas.

As análises descritivas e bivariadas foram realizadas no software IBM SPSS Statistics, versão 27.0, utilizando o módulo de amostras complexas. A análise multivaria-da (regressão de Poisson) foi conduzida no software R (versão 4.5.1) com o pacote *survey*.

Em ambas as análises, aplicou-se o peso de expan-são populacional (pesorake). Esse peso, fornecido na base de dados, foi calculado pelo próprio Vigitel através de um método de pós-estratificação (rake). O pesorake corrigiu simultaneamente a diferente probabilidade de seleção de cada indivíduo e as distorções amostrais decorrentes da não resposta e da cobertura, ajustando a amostra para que ela correspondesse às estimativas populacionais do Censo Demográfico para cada capital, segundo es-tratos de sexo, faixa etária e nível de escolaridade.

Considerou-se a associação com p-valor<0,050 como estatisticamente significativa.

## Resultados

A amostra deste estudo foi composta por 2.255 adultos residentes nas capitais da região Sul do Brasil. A prevalência ponderada de hipertensão autorrefe-rida para o conjunto das três capitais foi de 28,0% (IC95% 25,4; 30,8). Houve variação estatisticamente significativa entre os municípios. Porto Alegre apre-sentou a prevalência de 32,8% (IC95% 28,2; 37,8), e Florianópolis teve a menor prevalência, com 22,7% (IC95% 19,4; 26,5) (Tabela 1).

**Tabela 1:** Prevalência de hipertensão autorreferida e intervalo de confiança (IC95%), segundo capitais da região Sul. Curitiba, Florianópolis e Porto Alegre, 2023 (n=2.255)

Município^a^	N^b^	Prevalência de hipertensão (%) (IC95%)^c^
Curitiba	757	25,6 (22,0; 29,6)
Florianópolis	756	22,7 (19,4; 26,5)
Porto Alegre	742	32,8 (28,2; 37,8)
Total	2.255	28,0 (25,4; 30,8)

^a^Teste de independência de Rao-Scott (F) para a diferença estatística entre os municípios (F 5,68; p-valor 0,007); ^b^Os valores de (n) referem-se às contagens não ponderadas da amostra; ^c^As prevalências são ponderadas.

Observou-se predominância do sexo feminino (n=1.385; 61,4%) (Tabela 2). A faixa etária mais repre-sentada foi de 70 anos ou mais (n=468; 20,8%). A maior parte dos entrevistados relatou ser casado legalmente (n=870; 38,6%). Houve predominância de raça/cor bran-ca (n=1.724; 76,5%). A maior frequência foi de indivíduos com nível superior completo ou mais (n=941; 41,7%).

Na análise bivariada (Tabela 2), a prevalência de hipertensão aumentou progressivamente com a faixa etária (F 34,29; p-valor<0,001). O estado conjugal atual (F 14,8; p-valor<0,001) também mostrou associação estatisticamente significativa, sendo a hipertensão mais prevalente entre viúvos(as), com 58,8% (IC95% 47,4; 69,5). A prevalência de hipertensão diminuiu progressivamente com o aumento da escolaridade (F 21,24; p-valor<0,001).

**Tabela 2:** Prevalência de hipertensão, intervalo de confiança (IC95%) e análise bivariada, segundo características sociodemográficas. Curitiba, Florianópolis e Porto Alegre, 2023 (n=2.255)

Variável	Categoria	N^a^	Prevalência de hipertensão (%) (IC95%)^b^	p-valor^c^
**Sexo**	Masculino	870	28,1 (24,3; 32,3)	0,943
	Feminino	1.385	27,9 (24,5; 31,6)	
**Faixa etária (anos)**	18-29	262	4,4 (2,0; 9,2)	<0,001
	30-39	315	19,1 (12,9; 27,5)	
	40-49	381	23,3 (17,7; 30,1)	
	50-59	399	38,8 (32,8; 45,1)	
	60-69	430	48,2 (41,7; 54,9)	
	70+	468	66,3 (60,4; 71,7)	
**Estado conjugal atual**	Solteiro(a)	625	17,2 (13,1; 22,1)	<0,001
	Casado(a) legalmente	870	32,4 (28,5; 36,6)	
	União estável há mais de seis meses	317	29,3 (22,4; 37,2)	
	Viúvo(a)	244	58,8 (47,3; 69,5)	
	Separado(a) ou divorciado(a)	199	39,4 (29,8; 49,9)	
**Raça/cor**	Branca	1.724	28,7 (25,6; 31,9)	0,825
	Preta	129	28,2 (17,5; 42,1)	
	Amarela	22	32,9 (14,5; 58,6)	
	Parda	368	25,1 (19,8; 31,2)	
	Indígena	12	35,1 (11,5; 69,1)	
**Escolaridade**	Sem instrução/fundamental incompleto	201	53,1 (42,7; 63,2)	<0,001
	Fundamental completo	363	45,2 (37,8; 52,8)	
	Ensino médio completo	750	26,0 (21,7; 30,8)	
	Superior completo ou mais	941	20,0 (16,7; 23,9)	

^a^Os valores de (n) referem-se às contagens não ponderadas da amostra; ^b^As prevalências são ponderadas; ^c^Teste de independência de Rao-Scott (F).

Apresentaram-se os resultados da análi-se multivariada, ajustada para as covariáveis (Tabela 3). Após o ajuste, permaneceram como fatores associados independentemente à hipertensão: o aumento da faixa etária (RP 13,15; IC95% 5,93; 29,15 para ≥70 anos), a obesidade (RP 2,01; IC95% 1,70; 2,38) e a diabetes (RP 1,53; IC95% 1,31; 1,79).

**Tabela 3:** Razões de prevalência ajustadas (RP) e intervalos de confiança (IC95%) para hipertensão, segundo variáveis sociodemográficas, indicadores de saúde e estilo de vida. Curitiba, Florianópolis e Porto Alegre, 2023 (n=2.255)

Variável	Categoria	RP (IC95%)^a^	p-valor
**Sociodemográficas**			
Sexo	Masculino	1	
	Feminino	1,08 (0,91; 1,27)	0,377
Faixa etária (anos)	18-29	1	
	30-39	4,27 (1,83; 9,97)	<0,001
	40-49	4,74 (2,10; 10,68)	<0,001
	50-59	7,81 (3,54; 17,25)	<0,001
	60-69	9,24 (4,14; 20,61)	<0,001
	70+	13,15 (5,93; 29,15)	<0,001
Estado conjugal atual	Solteiro(a)	1	
	Casado(a) legalmente	0,91 (0,70; 1,17)	0,450
	União estável há mais de seis meses	1,09 (0,78; 1,52)	0,609
	Viúvo(a)	1,02 (0,76; 1,38)	0,901
	Separado(a) ou divorciado(a)	1,11 (0,81; 1,51)	0,531
**Raça/cor**	Branca	1	
	Preta	1,21 (0,79; 1,87)	0,378
	Amarela	1,46 (0,80; 2,65)	0,213
	Parda	0,91 (0,73; 1,12)	0,358
	Indígena	0,90 (0,49; 1,66)	0,743
Escolaridade	Sem instrução/fundamental incompleto	1	
	Fundamental completo	1,15 (0,91; 1,43)	0,237
	Ensino médio completo	0,99 (0,79; 1,25)	0,944
	Superior completo ou mais	0,79 (0,61; 1,01)	0,064
**Indicadores de saúde**			
Obesidade	Não obeso	1	
	Obeso	2,01 (1,70; 2,38)	<0,001
Diabetes	Não diabético	1	
	Diabético	1,53 (1,31; 1,79)	<0,001
**Estilo de vida**			
Sedentarismo	Não sedentário	1	
	Sedentário	1,13 (0,95; 1,35)	0,153
Tabagismo	Não fumante	1	
	Fumante	0,97 (0,75; 1,25)	0,808

^a^Ajustado para todas as variáveis listadas na tabela.

## Discussão

Neste estudo, foi possível identificar que a hiper-tensão entre adultos residentes nas capitais da região Sul do Brasil tem associações à faixa etária avançada, à menor escolaridade, à obesidade, ao sedentarismo e à diabetes. Contudo, a prevalência de hipertensão autorreferida entre adultos dessas capitais teve valor semelhante ao encontrado em demais trabalhos, com dados coletados também do Vigitel, que analisou o período de 2019 [11].

Verificaram-se limitações no estudo como o uso de dados secundários, autorreferidos, sujeitos a viés de memória, a incompletude de dados, a subnotifica-ção e a exclusão de indivíduos sem acesso telefônico, o que pode limitar o tamanho da amostra.

Observou-se maior prevalência de respostas entre o público feminino, porém não foram verificadas dife-renças estatisticamente significativas entre os sexos. Esse dado dialogou com o cenário epidemiológico global, no qual cerca de 600 milhões de mulheres sofrem com alteração de níveis pressóricos, condição indicada como a principal causa de mortalidade feminina [12].

A maior prevalência de hipertensão autorreferida concentrou-se no grupo etário de 70 anos ou mais. A idade configurou-se como fator de risco não modi-ficável, caracterizado pela probabilidade crescente de desenvolvimento de aumento da pressão ao longo do tempo, especialmente após a quinta década de vida. Esse aumento está ligado às alterações fisiológicas e hemodinâmicas decorrentes do envelhecimento [13], com uma projeção de crescimento de 7% na prevalência global entre 2015 e 2050 [14]. Logo, indivíduos mais velhos têm maior dificuldade de adotar novos hábitos e estabelecer o estilo de vida mais saudável, com limitações na compreensão do impacto e da gravidade da doença, o que prejudica a incorporação de práticas de autocuidado [15].

Quanto aos critérios modificáveis, foi identificada predominância de hipertensão em pessoas sem instrução ou com ensino fundamental incompleto, apontando a escolaridade como influência na desigualdade em saúde [16]. Observou-se que sujeitos com menor escolaridade têm mais vulnerabilidade para desenvolver hipertensão, evento que potencialmente está associado a piores condições socioeconômicas, podendo ocasionar dificuldade no acesso ao serviço de saúde [17].

Ainda sobre os hábitos de vida diários, considerados fatores modificáveis, como a prática de exercício físico, a alimentação saudável e a não utilização de tabaco e álcool, são as principais ações de controle não medicamentoso, pois auxiliam na melhora da saúde cardiovascular [18]. Portanto, a não adesão à atividade física ocasiona sedentarismo, que provoca aumento da gordura corporal e acúmulo no lúmen das veias e artérias, gerando arteriosclerose que eleva os níveis pressóricos [19]. Outra consequência é a obesidade, com prevalência entre as pessoas com hipertensão pesquisadas, que desencadeia elevação da pressão arterial decorrente de alterações nos mecanismos cardiometabólicos, amplia o débito cardíaco e consequentemente desregula a resistência cardiovascular sistêmica [20].

Constatou-se também menor prevalência de hipertensão entre indivíduos tabagistas que não foi estatisticamente significativa neste estudo. O tabagismo foi apontado como o segundo fator de risco que afeta diretamente as doenças cardiovasculares, além de contribuir para o desenvolvimento de dislipidemias e diabetes [21]. Visto que a diabetes gera desregulação da glicose no organismo, quando combinado o diagnóstico com hipertensão, e que tal característica se mostrou evidente no estudo [22], enfatizou-se o elevado risco de complicações como doença renal crônica, retinopatia e disfunções sexuais [23].

Em síntese, a prevalência de hipertensão na população do Sul do país foi de 28%, aliada a hábitos de vida como sedentarismo (correlacionado à obesidade, à alimentação e ao consumo de álcool e tabaco), escolaridade e fatores não modificáveis tal como a idade. Tais achados evidenciaram a importância de políticas públicas voltadas à promoção de hábitos saudáveis, o que forneceu ferramentas para a manutenção dos fatores de risco modificáveis e para a prevenção de agravos aos fatores não modificáveis.

## Data Availability

Os dados utilizados neste estudo são de domínio público, disponíveis no repositório oficial do Ministério da Saúde, no endereço: https://svs.aids.gov.br/download/Vigitel/. Os códigos para a construção de variáveis não disponíveis na base de dados original podem ser obtidos mediante solicitação ao autor correspondente.

## References

[1] 1 World Health Organization. Global report on hypertension: the race against a silent killer [Internet]. Geneva: WHO, 2023 4 nov. 2025. https://www.who.int/publications/i/item/9789240081062 .

[2] 2 Brasil. Ministério da Saúde. Vigitel Brasil 2019: vigilância de fatores de risco e proteção para doenças crônicas por inquérito telefônico. Brasília: Ministério da Saúde, 2020.

[3] 3 BRANDÃO, AA; RODRIGUES, CIS; BORTOLOTTO, LA; ARMSTRONG, AC; MULINARI, RA; FEITOSA, ADM et al. Diretriz Brasileira de Hipertensão Arterial - 2025. Arq Bras Cardiol. 2025;122(9):e20250624.10.36660/abc.20250624PMC1367114941294179

[4] 4 MALTA, DC; GOMES, CS; ANDRADE, FMD; PRATES, EJS; ALVES, FTA; OLIVEIRA, V et al. Hipertensão arterial em adultos no Brasil: análise da Pesquisa Nacional de Saúde, 2019. Rev Bras Epidemiol. 2021;24(Suppl 2):e210001.

[5] 5 BERNAL, RTI; ISER, BPM; MALTA, DC; CLARO, RM. Sistema de Vigilância de Fatores de Risco e Proteção para Doenças Crônicas por Inquérito Telefônico (Vigitel): mudança na metodologia de ponderação. Epidemiol Serv Saúde. 2017;26(4):701-712.10.5123/S1679-4974201700040000329211136

[6] 6 Instituto Brasileiro de Geografia e Estatística. Censo demográfico 2022: população por idade e sexo - resultados do universo [Internet]. Rio de Janeiro: IBGE, 2023 4 nov. 2025. https://biblioteca.ibge.gov.br/index.php/biblioteca-catalogo?view=detalhes&id=73102 .

[7] 7 Brasil. Ministério da Saúde. Secretaria de Vigilância em Saúde e Ambiente. Arquivos em download Vigitel [Internet]. Brasília: Ministério da Saúde, 2025 4 nov. 2025. https://svs.aids.gov.br/download/Vigitel .

[8] 8 World Health Organization. Obesity: preventing and managing the global epidemic: report of a WHO consultation [Internet]. Geneva: WHO, 2000 27 jun. 2025. https://apps.who.int/iris/handle/10665/42330 .11234459

[9] 9 RAO, JNK; SCOTT, AJ. On Chi-squared tests for multiway contingency tables with cell proportions estimated from survey data. Ann Stat. 1984;12(1):46-60.

[10] 10 BARROS, AJD; HIRAKATA, VN. Alternatives for logistic regression in cross-sectional studies: an empirical comparison of models that directly estimate the prevalence ratio. BMC Med Res Methodol. 2003;3:21.10.1186/1471-2288-3-21PMC52120014567763

[11] 11 QUADRA, MR; SCHÄFER, AA; SANTOS, LP; MELLER, FO. Fatores de risco modificáveis associados às doenças crônicas em adultos residentes nas capitais brasileiras e no Distrito Federal: Vigitel, 2019. Cad Saúde Colet. 2025;33(2):e33020163.

[12] 12 CHAPMAN, N; CHING, SM; KONRADI, AO; NUYT, AM; KHAN, T; TWUMASI-ANKRAH, B et al. Arterial hypertension in women: state of the art and knowledge gaps. Hypertension. 2023;80(6):1140-1149.10.1161/HYPERTENSIONAHA.122.2044836919603

[13] 13 DIAS, GS; COSTA, MCB; FERREIRA, TN; FERNANDES, VS; SILVA, LL; SANTANA JÚNIOR, LM et al. Fatores de risco associados à hipertensão arterial entre adultos no Brasil: uma revisão integrativa. Braz J Dev. 2021;7(1):963-978.

[14] 14 EGAN, BM; MATTIX-KRAMER, HJ; BASILE, JN; SUTHERLAND, SE. Managing hypertension in older adults. Curr Hypertens Rep. 2024;26:157-167.10.1007/s11906-023-01289-7PMC1090445138150080

[15] 15 QUEIROZ, MG; AQUINO, MLA; BRITO, ADL; MEDEIROS, CCM; SIMÕES, MOS; TEIXEIRA, A et al. Hipertensão arterial no idoso - doença prevalente nesta população: uma revisão integrativa. Braz J Dev. 2020;6(4):22590-22598.

[16] 16 ZACHER, M. Educational disparities in hypertension prevalence and blood pressure percentiles in the Health and Retirement Study. J Gerontol B Psychol Sci Soc Sci. 2023;78(9):1535-1544.10.1093/geronb/gbad084PMC1046153437279558

[17] 17 MALTA, DC; BERNAL, RTI; RIBEIRO, EG; MOREIRA, AD; FELISBINO-MENDES, MS; VELÁSQUEZ-MELÉNDEZ, JG. Hipertensão arterial e fatores associados: Pesquisa Nacional de Saúde, 2019. Rev Saude Publica. 2022;56:122.10.11606/s1518-8787.2022056004177PMC974966236629713

[18] 18 CHARCHAR, FJ; PRESTES, PR; MILLS, C; CHING, SM; NEUPANE, D; MARQUES, FZ et al. Lifestyle management of hypertension: International Society of Hypertension position paper endorsed by the World Hypertension League and European Society of Hypertension. J Hypertens. 2024;42(1):23-29.10.1097/HJH.0000000000003563PMC1071300737712135

[19] 19 ABREU, YL; ROCHA, F; TOLEDO, N; REBELO, LE. Estilo de vida como fator de risco para hipertensão arterial em adultos. Rev Hosp Univ Getulio Vargas. 2022;20(1):9-18.

[20] 20 CUNHA, CLP. Hipertensão induzida pela obesidade. Arq Bras Cardiol. 2023;120(7):e20230391.10.36660/abc.20230391PMC1042159637585897

[21] 21 WAGAI, GA; JEELANI, U; BEG, MA; ROMSHOO, GJ. Relationship between hypertension and smoking: a preliminary study in South Kashmiri population of J&K. J Family Med Prim Care. 2023;12(5):958-961.10.4103/jfmpc.jfmpc_2023_22PMC1033695737448933

[22] 22 Brasil. Ministério da Saúde. Secretaria de Atenção à Saúde. Estratégias para o cuidado da pessoa com doença crônica: hipertensão arterial sistêmica [Internet]. Brasília: Ministério da Saúde, 2013 4 nov. 2025. https://bvsms.saude.gov.br/bvs/publicacoes/estrategias_cuidado_pessoa_doenca_cronica.pdf .

[23] 23 HEZAM, AAM; SHAGHDAR, HBM; CHEN, L. The connection between hypertension and diabetes and their role in heart and kidney development. J Res Med Sci. 2024;29:22.10.4103/jrms.jrms_470_23PMC1116208738855561
